# Essentialism promotes children's inter-ethnic bias

**DOI:** 10.3389/fpsyg.2015.01180

**Published:** 2015-08-12

**Authors:** Gil Diesendruck, Roni Menahem

**Affiliations:** Department of Psychology, Bar-Ilan UniversityRamat-Gan, Israel

**Keywords:** essentialism, attitudes, social categories, children, ethnicity

## Abstract

The present study investigated the developmental foundation of the relation between social essentialism and attitudes. Forty-eight Jewish Israeli secular 6-year-olds were exposed to either a story emphasizing essentialism about ethnicity, or stories controlling for the salience of ethnicity or essentialism *per se*. After listening to a story, children's attitudes were assessed in a drawing and in an IAT task. Compared to the control conditions, children in the ethnic essentialism condition drew a Jewish and an Arab character as farther apart from each other, and the Jewish character with a more positive affect than the Arab character. Moreover, boys in the ethnic essentialism condition manifested a stronger bias in the IAT. These findings reveal an early link between essentialism and inter-group attitudes.

## Introduction

People essentialize certain social categories. In particular, people believe that certain social categories capture objective partitions of reality, are composed of homogenous members who share inherent and unique characteristics, and that membership in the category is biologically determined and developmentally stable (Rothbart and Taylor, 1992; Hirschfeld, 1996; Gelman, 2003). Already in the 50s, Allport (1954) argued that such a construal of social categories likely underlies, and promotes, intergroup prejudice. In fact, numerous studies have documented that social essentialism is linked to adults' tendencies to hold stereotypes, prejudice, and negative attitudes toward essentialized groups (Yzerbyt et al., 2001; Haslam et al., 2002; Leyens et al., 2003; Keller, 2005; Prentice and Miller, 2007; Williams and Eberhardt, 2008; Morton et al., 2009).

A number of proposals have been raised in the social psychological literature, attempting to explain the link between essentialism and attitudes. Among others, it has been suggested that dominance orientation (Sidanius and Pratto, 2001), group identification (Tajfel, 1982), and ideologies (Jost et al., 2003), may all serve as important catalysts of the relation. The goal of the present study was to shed light on this discussion, by examining whether the link between essentialism and inter-group attitudes is already manifest early in development. The rationale is that to the extent that this link is evident in young children, then that sets constraints on the plausible mechanisms accounting for it.

Developmental studies reveal that already prior to entering school, children hold both, essentialist beliefs about various social groups (e.g., Rhodes and Gelman, 2009; Kinzler and Dautel, 2012), and intergroup biases (e.g., Aboud, 2003; Abrams, 2011; Dunham et al., 2011). However, although the potential link between the two constructs has indeed been directly hypothesized (Bigler and Liben, 2007), so far it has been investigated only in correlational studies. In particular, children's beliefs about the constancy and stability of racial identity was found significantly related to both, the extent to which they assign positive traits to their ingroup and negative ones to their outgroup (Rutland et al., 2005), and the extent to which they hold racial stereotypes—primarily regarding outgroups (Pauker et al., 2010; see also Levy and Dweck, 1999). Evidently, it is impossible to draw conclusions about the directionality of the relation, and thus it is impossible to rule out whether, for instance, children who are more racially biased, “justify” their bias by adopting essentialist beliefs. The present study was designed to tackle this issue more directly by providing an experimental test of this link.

The social category investigated was one that has been proven highly significant for our target population; namely, ethnicity, manifested in Israel in terms of the distinction between Jews and Arabs. Studies have shown that by 4- or 5-years of age, Israeli Jewish children hold negative attitudes toward Arabs (Bar-Tal and Teichman, 2005), and start showing evidence of essentialist beliefs about ethnic categories (Birnbaum et al., 2010; Deeb et al., 2011). Given these findings, the present study investigated the relation between essentialism and attitudes, at the youngest age in which these phenomena seem to be clearly in place, namely, 6-year-olds.

The experimental method used in the study was adapted from ones previously used with adults (e.g., Keller, 2005). Specifically, we told children a story designed to prompt essentialist thinking about ethnicity, in that it incorporated a number of the component notions associated with essentialist beliefs, e.g., that members of different ethnic groups differ in many fundamental respects, and that ethnic membership is stable throughout development and inherited from one's biological parents. Control stories were included to rule out the possible effects of sheer exposure to essentialist notions, or the salience of information about ethnicity. After hearing their corresponding story, participants were given two tasks to measure their attitudes toward ethnic groups.

The first attitudes task was a drawing task, in which children were simply asked to draw a picture of a Jew and an Arab. Drawing tasks have been used to assess affect-related aspects of children's social relationships (Bombi and Pinto, 1994; Teichman, 2001), rendering both quantitative and qualitative indices. The present study focused on one quantitative and one qualitative measure, respectively, the distance between the two characters drawn, and the affective expression of the characters. Regarding the former, research on adults reveals that the physical distance between participants and members of an outgroup is indicative of participants' attitudes toward the outgroup (Kawakami et al., 2007; Goff et al., 2008). In a similar vein, research on children shows that the representation in drawings of the distance between the child and others is also indicative of the affective quality of the relationship (Bombi and Pinto, 1994; Holmes, 1997). Thus, we hypothesized that the stronger children's inter-ethnic bias, the farther apart they would draw the outgroup character (i.e., an Arab) from the ingroup one (a Jew). To ascertain the validity of this measure, we also assessed the correlation between children's responses in it, and a more standard measure of attitudes, namely the IAT. As for the affective expression of the characters, our hypothesis was that the stronger the bias, the more negatively they would draw the Arab character compared to the Jewish one.

The second attitudes task was the Child—Implicit Association Task (IAT) (Baron and Banaji, 2006). The IAT is a computerized task that assesses the speed with which a participant responds to pairings of concepts and evaluative terms. In the present study, the pairings were between pictures depicting exemplars of the two ethnic categories—Jews and Arabs—and positive (e.g., “good”) or negative (e.g., “bad”) evaluative words. The logic behind the IAT is that to the extent that people are biased such that they value ingroups positively and outgroups negatively, then they should respond faster to bias-consistent pairings (e.g., Jew-good or Arab-bad, for a Jewish participant) than to bias-inconsistent pairings (e.g., Jew-bad or Arab-good).

In general, our main hypotheses were that children exposed to the story endorsing ethnic essentialism—compared to those exposed to the control stories—would: (a) draw a Jewish and an Arab character as farther apart from each other; (b) draw an Arab character more negatively than a Jewish character, and (c) show a larger difference in response time between bias-consistent and bias-inconsistent trials in the IAT.

## Methods

### Participants

Participants in the present study were 48 secular Jewish Israeli 6-year-olds (*M* = 6 years 6 months, range = 5.9–7.1; 24 girls). All children lived in cities consisting almost exclusively of Jews, and attended exclusively Jewish non-religious public schools. Only children with signed parental permission participated. Children received a small gift in gratitude for their participation.

### Design

Participants were randomly assigned to one of three between-subjects conditions, defined by the “priming story” they were exposed to: Ethnic Essentialism, Ethnic Mention, and Animal Essentialism (*n* = 16, per condition). There were no significant differences in the mean ages or gender distribution among conditions.

### Materials

All materials and procedures were approved by the Ethics Committee of the Department of Psychology.

#### Priming stories

Three different stories were elaborated: a target story and two “control” stories. The target story was the *Ethnic Essentialism* story. The story emphasized various aspects of essentialism, such as the notions that ethnicity is inherited and stable throughout development, and that members of ethnic categories are homogenous and distinct from members of another ethnic category. The story described two boys, a Jew and an Arab, in counterbalanced order of presentation across participants. The *Ethnic Mention* story was elaborated to provide a control for the salience of ethnicity in the target story. The story described a boy—with no defined ethnicity—who while searching for his lost dog in a park, encountered a number of people whose ethnic membership was explicitly described (i.e., “Jew” and “Arab”). Thus, in this story, ethnic labels were mentioned the same number of times (8 each ethnicity) as in the Ethnic Essentialism story (see the Appendix for these two stories). The *Animal Essentialism* story was elaborated to provide a control for the sheer triggering of essentialism. The story had the same structure as the Ethnic Essentialism story, but instead of describing the lives of two boys from different ethnicities, it described the lives of two animals from different species (a giraffe and an elephant).

As a manipulation check, we read the Ethnic Essentialism and the Ethnic Mention stories to another group of 20 six-year-olds (10 each), and asked them a series of questions assessing their beliefs about components of ethnic essentialism expressed in the Ethnic Essentialism story. The questions were taken from the Essentialism Components Questionnaire (ECQ; Deeb et al., 2011), and had to do with: (1) the *distinctiveness* of Jews and Arabs—i.e., how differently Jews and Arabs think, behave, and look (1 = not all, to 4 = a lot), and (2) the *inheritability* of ethnic membership—i.e., if it is possible for someone born to parents of ethnicity A (e.g., Arab) to become someone from ethnicity B (e.g., Jew), and if it is possible for a mother from ethnicity A (e.g., Arab) to give birth to a baby who is from ethnicity B (e.g., Jew) (1 = very possible, to 4 = completely impossible). Confirming the manipulation, a MANOVA with the scores on these two components entered as dependent-variables revealed that children who heard the Ethnic Essentialism story had a higher overall essentialism score (*M* = 3.28, *SD* = 0.37) than those who heard the Ethnic Mention story (*M* = 2.69, *SD* = 0.45), *F*_(1, 17)_ = 5.14, *p* < 0.05, η^2^ = 0.38. Interestingly, although component type did not interact with story type, the largest difference between stories regarded the inheritability component (*M* = 4.00, *SD* = 0.00; and *M* = 3.18, *SD* = 1.08, for Ethnic Essentialism and Ethnic Mention, respectively).

#### Drawing task

Each participant was given a white A4 sheet of paper and 6 colored pencils, and was asked to draw a Jew and an Arab (in counterbalanced order across participants). Two measures were coded for analyses by two coders blind to the Priming Story children had heard. The first was the distance in centimeters between the two characters, measured with a ruler from the closest points of each character. The second measure was the affect of each of the characters, as manifested in their facial expressions. This variable was coded as expressing positive (3), neutral (2), or negative (1) affect, and agreement between coders was perfect. The dependent measures were the affective scores regarding Arab (affect-toward-Arab) and Jewish (affect-toward-Jew) characters.

#### IAT task

**Figure 1:**
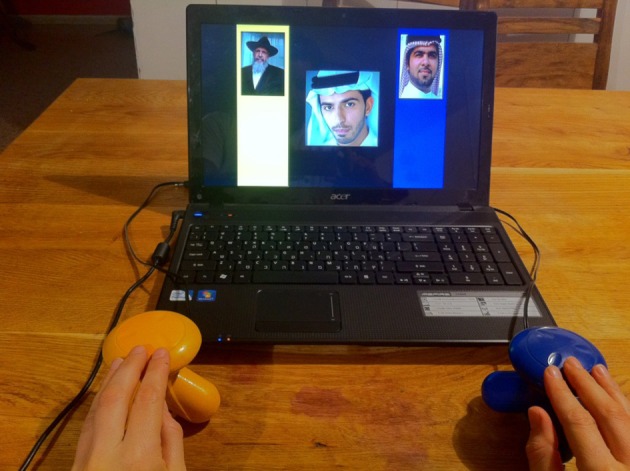
**Picture of the experimental set-up for the IAT**.

Each participant completed a version of the Child IAT, using ethnicity rather than race as a social category (Baron and Banaji, 2006). The task was presented to children as a “computer game,” in which after seeing pictures on a laptop screen or hearing certain words, they would have to press two different buttons as quickly as possible. To facilitate children's performance, they responded by pressing two large mouse-like buttons—one blue and one yellow—connected to the laptop computer (see Figure 1). Moreover, the laptop screen was marked with two distinct frames on each side—one blue and one yellow—matching the buttons on its side.

The pictures used for the task were of stereotypically dressed Jewish and Arab men with neutral or positive affective expressions (equally distributed between the two ethnicities). Jewish men were all orthodox, thus wearing a yarmulke, a black hat, or religious attire. Arab men were all portrayed wearing a head shawl (*kaffiah*). Four different pictures of each ethnicity were displayed throughout the task. The pictures were all males because the visual distinction between Jewish and Arab women is more ambiguous than between men. The positive evaluative words used were: *fun, good, happy*, and *nice*; the negative ones were: *disgusting, bad, angry*, and *mean*. The words were pre-recorded by a female adult, and children heard them via headphones, which they wore during the entire IAT task. All words are highly familiar to children this age.

The structure of the IAT task was identical to the one in Baron and Banaji (2006), and included 7 stages. In all stages, the move from one trial to the next occurred only after children pressed the correct button. Stage 1 was a training stage for the sorting of the pictures. Children were told that they would be seeing pictures of either Arabs or Jews in the center of the screen, and that they were to press the blue button as quickly as they could when the picture was of an Arab, and the yellow button when the picture was of a Jew. To help children remember the rule, in this stage, small pictures of an Arab and a Jew appeared on their corresponding halves of the screen. Stage 1 consisted of 20 trials, half of each kind, presented in random order. Children indeed succeeded in identifying the ethnicities of the pictures quite well. Stage 2 consisted of a training for the sorting of the evaluative words. Children were told that they would be hearing words through the headphones, some of them good and some of them bad. Children were instructed to press the blue button if they heard a bad word, and the yellow button if they heard a good word. To remind children of the instructions in this stage, small pictures of a sad and a happy “Smiley” faces appeared on their corresponding halves of the screen. Stage 2 too consisted of 20 trials, half of each kind, presented in random order.

Stage 3 required children to respond to the two types of stimuli—pictures and words—by pressing the same buttons they had been trained on in Stages 1 and 2. Namely, children were told that they would either see a picture of an Arab or a Jew, or they would hear a bad or a good word. If the picture was of an Arab or the word was bad, they had to press the blue button. If the picture was of a Jew or the word was good, they had to press the yellow button. Stage 3 consisted of 20 trials, a quarter of each kind (5 pictures of Jews, 5 of Arabs; 5 positive words, 5 negative), presented in random order. Stage 4 constituted one of the critical test stages, in which the strength of the associations between Jew-good and Arab-bad was assessed. This stage was identical to Stage 3, but consisted of 40 trials, a quarter of each kind, presented in random order.

In Stage 5, children were trained on the reversed association to the one displayed in the previous stages. The stage was identical to Stage 2 described above, but this time children were instructed to press the blue button (i.e., the same button for “Arabs”) when they heard good words, and the yellow button (i.e., the same button for “Jews”) when they heard bad words. Stage 5 consisted of 20 trials, half of each kind, presented in random order. Stages 6 (training) and 7 (test) were identical to Stages 4 and 5, except now the same buttons had to be pressed for inconsistent pairings (i.e., blue button for either Arab or good, yellow button for either Jew or bad).

Half of the participants performed the IAT task in the order of the stages described above. In other words, they were first trained and tested in pairings of pictures and evaluative words consistent with an ethnic bias, and then trained and tested on inconsistent pairings. For the other half of the participants, the order of stages was reversed, such that they were first trained and tested on inconsistent-pairings, and then on consistent ones. Specifically, this latter group of participants performed Stages 5–7 before Stages 2–4.

The dependent measure was a D-score (Greenwald et al., 2003), consisting of the weighted average of: (1) the difference between the average response time to inconsistent-pairings test trials (Stage 7) and the average response time to consistent-pairings test trials (Stage 4), divided by the pooled standard deviation in the pertinent stages, and (2) the corresponding difference in the respective training stages (Stages 6 and 3). Following the interpretation of previous research on the Child-IAT (Baron and Banaji, 2006), the higher the D-score, the longer it took participants to respond to inconsistent than to consistent pairings, and thus the stronger the intergroup bias.

### Procedure

Children were tested individually by an experimenter in a quiet room in their school. All participants went through the same sequence of tasks: (1) Priming story, (2) Drawing task, and (3) IAT. At the end of all tasks, the experimenter engaged the child in a brief informal conversation about how Jews and Arabs actually do live together in many places, work together, and have similar interests and activities.

## Results

### Preliminary analyses of control conditions

Given that our hypotheses referred to potential differences between the Ethnic Essentialism story and the two Control stories, prior to the analyses addressing the hypotheses, we compared children's responses in the two Control stories only. ANOVAs on the four main dependent-measures, i.e., distance, affect-toward-Arab, and affect-toward-Jew in the Drawing task, and D-score in the IAT, revealed no significant differences between the Ethnic Mention and the Animal Essentialism stories (on distance: *M* = 4.56, *SD* = 3.57, and *M* = 3.69, *SD* = 3.00; on affect-toward-Arab: *M* = 2.87, *SD* = 0.35, and *M* = 2.75, *SD* = 0.45; on affect-toward-Jew: *M* = 2.87, *SD* = 0.35, and *M* = 2.76, *SD* = 0.44; and on D-scores: *M* = 0.00, *SD* = 0.14, and *M* = −0.05, *SD* = 0.17; for Ethnic Mention and Animal Essentialism, respectively, all *ps* > 0.2). For this reason, in the main analyses we compared the Ethnic Essentialism story to the two Control stories combined.

### Drawing task

#### Distance

Due to experimenter's error, one child's drawing (from the Animal Essentialism condition) was misplaced, and so distance was analyzed for 47 children. An ANOVA with Priming story (Ethnic Essentialism, Controls), and Gender as between-subjects factors, using the distance between characters as the dependent measure revealed only a significant effect of Priming story, *F*_(1, 43)_ = 4.24, *p* < 0.05, η^2^ = 0.09. Namely, children exposed to the Ethnic Essentialism story (*M* = 6.51, *SD* = 4.45) drew the two characters as farther apart than did children exposed to the Control stories (*M* = 4.14, *SD* = 3.28).

#### Affect

**Figure 2:**
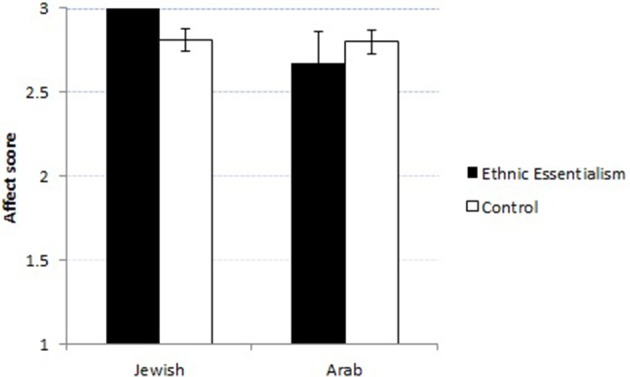
**Affective bias as a function of Priming story and character ethnicity**. 1, negative; 2, neutral; 3, positive. Error bars indicate SEs.

A repeated-measures ANOVA with Priming story (Ethnic Essentialism, Controls) and Gender as between-subjects factors, and Character (Arab, Jew) as within-subjects factor, using the affect scores as the dependent measures, revealed a significant effect of Character, *F*_(1, 43)_ = 4.73, *p* < 0.05, η^2^ = 0.1, and a significant interaction between Character and Priming story, *F*_(1, 43)_ = 4.73, *p* < 0.05, η^2^ = 0.1. Follow-up *t*-tests revealed that whereas Priming story did not have a significant effect on affect-toward-Arab (*p* > 0.5), children exposed to the Ethnic Essentialism story had significantly more positive affect-toward-Jew scores than those exposed to the Control stories, *t*_(31)_ = 2.68, *p* < 0.05 (see Figure 2).

### IAT task

Greenwald et al. (2003) recommended dropping from analyses adult participants who responded faster than in 300 ms on at least 10% of the trials—i.e., participants who seemed to be responding before fully processing the stimuli. Applying this criterion would have led to dropping 25% of our initial sample, and so we set the criterion at 15% of trials instead, leaving us with the sample of 48 children. An ANOVA with Priming story (Ethnic Essentialism, Controls), and Gender as between-subjects factors on participants' D-scores revealed a significant effect of Gender, *F*_(1, 44)_ = 13.89, *p* < 0.005, η^2^ = 0.24, and a significant interaction between Gender and Priming story, *F*_(1, 44)_ = 8.86, *p* < 0.01, η^2^ = 0.17. Follow-up ANOVAs on each Gender revealed that the effect of Priming story was significant only among boys, *F*_(1, 22)_ = 4.66, *p* < 0.05, η^2^ = 0.18, such that boys' D-score was higher after hearing the Ethnic Essentialism story (*M* = 0.13, *SD* = 0.17) than the Control stories (*M* = −0.01, *SD* = 0.14). This pattern did not seem to result from an irregular distribution of participants: 7 of the 8 boys who heard the Ethnic Essentialism story had positive D-scores, compared to 9 of the 16 who heard the Control stories. [An ANOVA identical to the one described above using the 10% criterion, also revealed a significant interaction between Priming story and Gender, *F*_(1, 33)_ = 9.52, *p* < 0.005, which derived from the fact that Priming story only affected boys, *F*_(1, 16)_ = 9.63, *p* < 0.01.]

### Relation between measures

In a final analysis, we looked at the relation between the two continuous measures used in the different tasks, i.e., distance in centimeters and D-score. Given the findings from the IAT, we conducted correlations separately for girls and boys. These analyses revealed that the correlation was significant only among boys (*r* = 0.51, *p* < 0.05). Namely, the farther apart boys drew the Jewish and Arab characters, the stronger was their implicit ethnic bias. This finding suggests that these two measures indeed tapped onto children's ethnic attitudes.

## Discussion

The present study revealed that experimentally reinforcing an essentialist construal of ethnicity influenced Israeli Jewish 6-year-olds' attitudes toward ethnicity. This effect was found beyond simply making ethnicity or essentialism salient, and in very distinct measures.

In a drawing task, children drew Jewish and Arab characters as farther apart from each other after hearing the Ethnic Essentialism story, than after hearing control stories. Thus, consistent with adults' responses to physical distance (Kawakami et al., 2007; Goff et al., 2008), and children's representations of social distance (Bombi and Pinto, 1994; Holmes, 1997), children who heard an essentialism-enforcing story represented Jews and Arabs as distant from each other. Although the assessment here was of children's depictions of distance, it is possible that, as in adults, there would be a correspondence between this represented distance, and the enactment of distance in real-life encounters (e.g., Williams and Eberhardt, 2008; Morton et al., 2009).

The findings regarding the effect of story on the affective expression of the drawn characters corroborated the above findings on distance. Namely, children who heard the Ethnic Essentialism story showed a stronger affective bias in favor of their ingroup than children who heard the Control stories. One of the main questions in the field of intergroup cognition has to do with whether the formation and conceptualization of social categories is driven by a need to affiliate with those who are like oneself, or to avoid those who are unlike oneself (e.g., Cosmides et al., 2003). Putting it differently, are intergroup biases driven by a positive bias toward ingroups or a negative bias toward outgroups (or both)? Studies on children's racial attitudes indicate that ingroup favoritism may developmentally precede outgroup derogation (Aboud, 2003; Buttelmann and Böhm, 2014). Consistent with this pattern, we found that compared to the Control conditions, the affective bias generated by the Ethnic Essentialism story appeared to result primarily from more positive valuation of ingroups, without a corresponding negative valuation of outgroups.

The effect of the essentialism story on IAT responses was found only among boys. Again, those exposed to the Ethnic Essentialism story showed a stronger implicit bias than those exposed to the Control stories. One possible reason for the fact that the effect of the essentialism manipulation on IAT performance was not as comprehensive is that, given the order of the tasks—the IAT coming after the drawing task—the effect of the story might have waned by the time children performed the IAT. Evidently this still cannot explain why this decline would have occurred more strongly for girls than for boys—though given the particularity of this finding, one should exercise caution in interpreting it. Nonetheless, one speculative explanation for this gender effect is that males are arguably more prone to intergroup biases than females (Navarrete et al., 2010), a pattern that seems to be manifested even among children (Geary et al., 2003). In fact, recent studies reveal that boys may have stronger intergroup biases than girls (Fehr et al., 2008; Buttelmann and Böhm, 2014; Benozio and Diesendruck, 2015). In general, it is important to remark that the IAT scores were also informative, in that they provided corroboration for the validity of the distance measure.

More generally, one theoretical implication of the present findings is that whatever drives the relation between essentialism and attitudes, it is already present among 6-year-olds. For instance, it has been argued that the relation may be driven by dominance orientation (Sidanius and Pratto, 2001), group identification (Tajfel, 1982), and political views (Jost et al., 2003). Developmental psychologists have been examining the emergence of some of these motivational constructs among children (Nesdale et al., 2005; Baron and Banaji, 2009; Killen et al., 2013), and thus a future integration of such factors to the study of essentialism and attitudes should be fruitful. Alternatively, others have argued that the relation between essentialism and intergroup bias derives from primary evolutionary motives (Cosmides et al., 2003). Future studies should attempt to address this alternative by examining even younger children than the ones tested here (see for instance, Buttelmann et al., 2013; Powell and Spelke, 2013), or assessing children in different cultures. In this latter regard, it is noteworthy that the extent to which children essentialize particular social categories (Diesendruck et al., 2013), and the extent to which they manifest implicit and explicit intergroup attitudes (Pauker et al., 2010), likely differ across cultures.

Finally, a practical implication of these findings is that if one is interested in reducing the emergence of negative attitudes toward particular groups, then it might be fruitful to target interventions at the primary—essentialist—beliefs potentially catalyzing such attitudes, already early on in development. The optimistic outlook in this regard is that there are strategies that have proven to work in reducing young children's social essentialism. In particular, showing children that people's characteristics fall within continua rather than being all-or-none (Master et al., 2012), using non-generic language to describe people (Cimpian and Markman, 2011; Rhodes et al., 2012), and having children interact daily and collaboratively with members of another group (Rutland et al., 2005; Deeb et al., 2011), have all been shown to reduce essentialist-like beliefs. The experimental link between essentialism and attitudes demonstrated here provides substantive support for such interventions.

## Author contributions

GD and RM designed the study. RM conducted the study. GD and RM analyzed the data. GD wrote the first draft of the paper. GD and RM commented on, and approved, the final version of the manuscript.

### Conflict of interest statement

The authors declare that the research was conducted in the absence of any commercial or financial relationships that could be construed as a potential conflict of interest.

## Appendix

### Priming stories

#### Ethnic essentialism

“I'm going to tell you a story about two boys: Hasan and Eyal. Hasan was born to Arab parents and therefore he is also an Arab. He grew up in a village where all the people are Arabs. In Hasan's village, everybody speaks Arabic and do the same things. For instance, every Friday afternoon they go to the village's mosque to pray together to the God of the Arabs, and the adult men wear a keffiah on their heads. Hasan already wants to be a grown-up so he too can wear a keffiah. Hasan loves his family, especially his two older brothers, who got married to Arab women and they now have cute children, who are Arabs of course, and with whom Hasan likes to play. Hasan dreams that 1 day he too will get married, have children, and everybody will keep growing together as Arabs, speaking Arabic, and living in the Arab village.

In another city lives Eyal. Eyal was born to Jewish parents and therefore he is Jewish. Only Jews live in Eyal's city. They don't speak Arabic, don't go to mosques, and don't wear keffiahs on their heads. In Eyal's city, everybody speaks Hebrew, and they go to the synagogue to pray to the God of the Jews. Eyal loves his family. Eyal's mother is pregnant and soon he is going to have a little sister. Of course she is also going to be Jewish. Eyal dreams that he and his sister will grow up to be close siblings, and they too will have Jewish children, and they will all grow up together as Jews, speaking Hebrew, and living in the Jewish city.”

#### Ethnic mention

“One day, a boy went out to look for his lost dog. The boy went out with his mother to the park next to his house. The first man they encountered in the park was a Jew. The boy asked the Jewish man: ‘Excuse me sir, did you see a little black and white dog walking alone?’ The Jewish man thought for a second and then replied: ‘No, I didn't. Perhaps you should ask that Arab man who is eating an apple.’ The boy and his mother left the Jewish man and went to ask the Arab man who was eating the apple. ‘Excuse me sir, do you know where I can find me dog? He is black and white.’ The Arab man thought and then said: ‘I'm sorry but I didn't see a black and white dog. May be you can ask that Arab woman sitting over there reading the newspaper.’ The boy and his mother left the Arab man and went toward the Arab woman who was sitting reading the newspaper. The boy asked her: ‘Excuse me ma'm, I'm looking for my dog. Did you see him passing by?’ The Arab woman looked at the boy and said: ‘I haven't seen any dogs here. You should ask that Jewish woman who is pushing a stroller.’ The boy left the Arab woman and went running toward the Jewish woman who was pushing a stroller. ‘Excuse me miss, perhaps you have seen my dog?’ ‘No, I haven't,’ answered the Jewish woman. At this point, the boy's mother told him: ‘We should go home. Our dog is very smart, so perhaps he found his way back.’ The mother and the boy left the Jewish woman and went back home. When they got there, they found the dog waiting by the door. The boy gave his dog a big hug, and he was very happy. He said: ‘From now on, we will always go out together.’”
